# The Adoption of Cloud Computing in the Field of Genomics Research: The Influence of Ethical and Legal Issues

**DOI:** 10.1371/journal.pone.0164347

**Published:** 2016-10-18

**Authors:** Kathleen Charlebois, Nicole Palmour, Bartha Maria Knoppers

**Affiliations:** Centre of Genomics and Policy, Faculty of Medicine/Department of Human Genetics, McGill University, Montréal, Québec, Canada; University of Rochester, UNITED STATES

## Abstract

This study aims to understand the influence of the ethical and legal issues on cloud computing adoption in the field of genomics research. To do so, we adapted Diffusion of Innovation (DoI) theory to enable understanding of how key stakeholders manage the various ethical and legal issues they encounter when adopting cloud computing. Twenty semi-structured interviews were conducted with genomics researchers, patient advocates and cloud service providers. Thematic analysis generated five major themes: 1) Getting comfortable with cloud computing; 2) Weighing the advantages and the risks of cloud computing; 3) Reconciling cloud computing with data privacy; 4) Maintaining trust and 5) Anticipating the cloud by creating the conditions for cloud adoption. Our analysis highlights the tendency among genomics researchers to gradually adopt cloud technology. Efforts made by cloud service providers to promote cloud computing adoption are confronted by researchers’ perpetual cost and security concerns, along with a lack of familiarity with the technology. Further underlying those fears are researchers’ legal responsibility with respect to the data that is stored on the cloud. Alternative consent mechanisms aimed at increasing patients’ control over the use of their data also provide a means to circumvent various institutional and jurisdictional hurdles that restrict access by creating siloed databases. However, the risk of creating new, cloud-based silos may run counter to the goal in genomics research to increase data sharing on a global scale.

## Introduction

Cloud computing facilitates the storage and management of large amounts of data, but can also serve as a possible instrument of surveillance. How cloud computing is perceived, understood and adopted varies depending on the needs and preferences of a set of stakeholders in a particular field [1]. In genomics research, cloud computing technology provides a way for researchers to enhance their capacity to store and share data, save time and reduce costs of data sharing [2, 3]. With next-generation sequencing yielding unprecedented amounts of data, the ability of cloud computing to search for common patterns and to generalize results will accelerate the development of treatments and diagnostic tools.

Harnessing the potential of cloud computing however, may well depend on how the ethical, legal and security challenges are addressed [3–6]. As researchers, patient advocates and cloud service providers are faced with these challenges, they struggle to address them without impeding data sharing. One major challenge relates to the risk of a security breach in a cloud computing system [3]. The risk of a data breach is further exacerbated as data are transferred by cloud service providers between data centers situated in different jurisdictions [4, 5]. As a tool in genomics research, cloud computing must also address privacy issues, especially that of possibly identifying an individual.

One definition of cloud computing applied to the field of genomics research is the following: “a scalable service where genetic sequence information is stored and processed virtually usually via networked, large-scale data centers accessible remotely through various clients and platforms over the Internet”[4]. More specifically, cloud technology is divided into three major service arrangements: 1) infrastructure (Amazon Web Services, Google), 2) platforms (ex.: Globus Genomics) and 3) software (ex.: Dropbox)[4–7]. Cloud services also comprise three deployment models (public, private or hybrid) that vary depending on the extent to which they are freely accessible [4–7]. Decisions over the type of services and deployment models influence the form cloud computing adoption takes and may reflect how varying ethical, legal and social challenges are managed.

The overarching aim is to achieve a balance between data sharing and ensuring the privacy of genomic data. Recommendations to that end range from the need for researchers to gain awareness of how cloud service providers treat data [4] to the establishment of endeavors focused on clarifying such issues [3]. Regarding the latter, initiatives, such as the Cancer Genome Collaboratory as well as the Global Alliance for Genomics and Health (GA4GH), have been established with the aim of facilitating the development of powerful computational tools that would enable research to be conducted on large data sets, while addressing the ethical, social and privacy issues that are thought to inhibit cloud computing adoption and therefore, data sharing [8, 9].

Examining how the issues surrounding the adoption of cloud computing could provide clarifications on how best to ensure that cloud computing is used responsibly. Thus, our research objective is to identify the various ethical, privacy and security issues facing the adoption of cloud computing in genomics research. Our study firstly examines how these issues shape cloud computing adoption and use in the field of genomics research and secondly, seeks to understand how they are managed by genomics researchers, patient advocates and cloud services providers.

## Framing Cloud Computing Adoption in Genomics Research

In order to understand how ethical, social and legal issues are managed by genomics researchers, patient advocates and cloud service providers and how this shapes cloud computing adoption, we drew on Diffusion of Innovation theory, and adapted it to consider the ethical, privacy and security issues arising from the adoption and use of cloud technology in genomics research. Diffusion of innovation theory [10] (DoI) posits that decisions regarding the adoption of a new technology revolve around five dimensions: relative advantage, compatibility, complexity, trialability and observability [10]. Other models add the dimensions of trust and security, which pertain to ethics and privacy [11, 12].

### Relative advantage

Relative advantage refers to the “degree to which using an innovation is perceived as making one better off than otherwise” [10, 13]. The extent to which an innovation is viewed as an improvement to already existing technologies may influence the adoption of a novel technology, as well as its use. According to studies that focus on cloud computing, cloud technology is attributed to facilitating data sharing among researchers as well as the storage of data generated following next-generation sequencing at a low cost. Other advantages include its scalability and in particular, its cost-effectiveness with respect to the amount of data it can store [4, 5].

### Compatibility

Compatibility refers to the degree to which an innovation is perceived to be consistent with internal organizational and information systems environments, as well as with already held values and beliefs prior to adopting the technology [10, 13]. The degree of compatibility with pre-existing computer systems influences the adoption of cloud computing. Compatibility also speaks to how cloud technology is confronted by already existing norms and values. The latter are embedded in various policies and regulations surrounding data privacy, be they institutional or jurisdictional. Researchers’ familiarity with the ethical, privacy and security issues pertaining to cloud computing play a role in their assessment and adoption of this technology. Compatibility rests on efforts to align or adjust cloud technology with already existing institutional and information technology (IT) environments as well as norms and values (as embedded in policies and regulations), or vice versa.

### Complexity

Complexity discusses the degree to which using an innovation is perceived as a difficult task or complex, and its ease of use or necessitating additional training or support [10, 13]. Shaping perceptions over the usability of a novel technology is the perceived difficulty in learning how to use it [11]. This, in turn, shapes the manner in which cloud technology is adopted, in particular, the type of deployment models potential users will wish to install.

### Trialability-trust

While trialability is defined as the possibility for a potential user to try out a novel technology before adopting it[10, 13], other uses of DoI theory focus instead on issues of security and trust [11, 12]. Indeed, trust between potential users and cloud service providers is required for a potential user to be willing to try out a novel technology before adoption. Trust is defined as the extent to which a party relinquishes control to another party on the belief that it will fulfill tasks and responsibilities it values [14]. Shaping cloud computing adoption are the relationships between genomics researchers, cloud service providers and patient advocates. Of particular interest is the relationship between cloud service providers and customers. Cloud service providers tend to be perceived as lacking transparency as to how they go about ensuring the security and privacy of the cloud, as it pertains to sensitive data that may identify a patient [3, 4, 15]. Issues of trust and transparency center on not only the nature (and power equilibrium) in contractual agreements, but also monitoring by the customer of how their cloud service provider stores and secures the data.

### Observability

Observability refers to the degree to which cloud computing is considered a must within a certain field [10, 12]. What prompts potential users to adopt a technology may be influenced by its reputation within their field, as well as within society. Uses of cloud computing in other fields, such as the financial sector, may be exemplars of success [16] and may compel potential users to adopt a novel technology. In the face of ongoing security and privacy concerns in the adoption and use of cloud computing, efforts are being made to augment public confidence in the field [3, 4, 6, 17].

## Materials and Methods

### Research Design

Our research project consists of a single case study[18]. Given our aim to study a phenomenon, cloud computing adoption, in a specific context, genomics research, a case study approach was deemed most appropriate. The unit of analysis includes the experiences, direct and indirect, with adoption and use cloud computing as a research tool. We thus sought to trace the process through which genomics researchers (GRs), patient advocates (PAs) and cloud service providers (CSPs) consider ethical, privacy and security issues when adopting cloud technology in the context of genomics research.

### Sampling and recruitment

**Fig 1:**
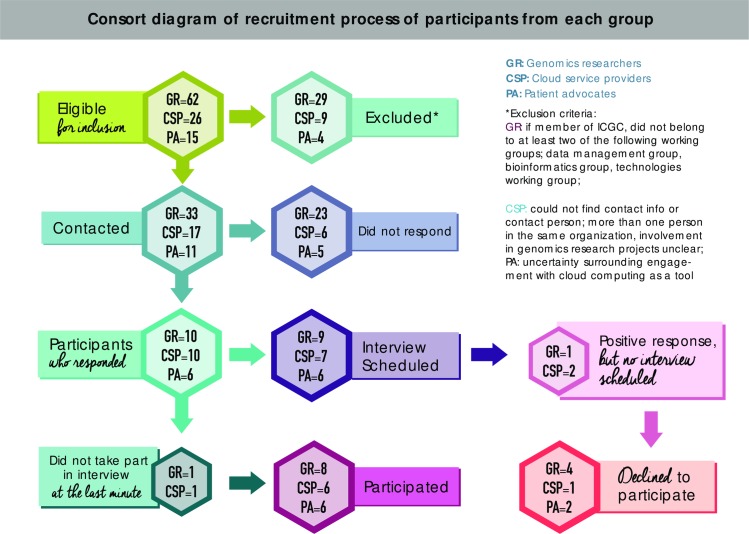
Consort diagram of recruitment process of participants from each group.

A combination of sampling strategies was used to recruit participants. A convenience sampling frame served as a starting point, after having contacted known cloud computing providers, users and patient advocacy groups with data repositories. Participants were mainly drawn from web sites of leading genomic consortia in the field of genomics research, namely the Global Alliance for Genomics and Health and the Cancer Genome Collaboratory. A purposeful sampling strategy was employed to ensure that potential participants were selected according to our research objective [19]. On that basis, criteria were used to select potential participants from each group according to our research aims (see Table 1). We focused on participants most closely associated with the innovation and integration process in cancer genomics. We also asked the respondents to suggest other names and utilized a snowballing strategy [19] (Fig 1).

**Table 1:** Selection criteria for recruitment of participants.

Genomics researchers	Patient advocates	Cloud service providers
Inclusion criteria		
Whether participant is author or co-author in studies in cancer and/or genomics research	Involvement (past or present) in genomic research projects using cloud computing as a tool (this involvement may include not only actually using cloud computing but may also include those who have used cloud computing, are considering cloud computing or considered using it in the past)	Engagement (direct or indirect) with members of the genomic research community-Consider themselves familiar with legal/ethical issues regarding cloud computing
Membership in initiatives aimed at cloud computing in genomics research, such as the Global Alliance; International Cancer Genome Consortium; Cancer Genome Collaboratory	Consider themselves to be engaged (direct or indirect; past or present) with cloud service providers	Role in health sector and/or IT sector management within their organization
Self-identify as being familiar with cloud computing, either direct or indirect, as a genomics research tool	Membership in initiatives partially aimed at cloud computing in genomics research, such as the Global Alliance; International Cancer Genome Consortium; P3G; ISBER; Cancer Genome Collaboratory	Self-identify as being familiar with their organization’s role in providing cloud computing to genomic researchers and patient advocates in genomic research
		Membership in initiatives aimed at cloud computing in genomics research, such as the Global Alliance; International Cancer Genome Consortium; P3G; ISBER; Cancer Genome Collaboratory
Exclusion criteria		
If member of ICGC, did not belong to at least two of the following working groups; data management group, bioinformatics group, technologies working group	Uncertainty surrounding engagement with cloud computing as a tool	Could not find contact info or contact person; more than one person in the same organization, involvement in genomics research projects unclear

### Data Collection

Semi-structured interviews consisting of open-ended questions were used to collect data and generate in-depth insight from CSP and cloud client communities and representatives, research communities (researchers, bioinformaticians, physicians, clinician-researchers, etc…) and representatives of the patient donor communities on the use of genomic cloud computing to store, analyze and share genomic data. Interviews were conducted until no new changes were made to the codebook, which served as a basis for data saturation [20]. Data saturation was assessed using a saturation table that was designed around the codebook and included a summary of the content [21, 22] and in part on Francis et al.’s approach [23]. Our initial sample size consisted of sixteen participants, as a way of ensuring an even group distribution [24]. We thus attempted to ensure this even distribution as much as possible, between the three groups, although a larger number of genomics researchers were interviewed (see Table 2). Ultimately, we achieved data saturation after conducting twenty interviews.

**Table 2:** Distribution of interviews conducted according to group of participants.

Group of participants	Interviews conducted	Country of origin			
		Canada	European Union (Germany, Spain, UK)	US East	US West
Genomics researchers	8	1	3	2	2
Cloud service providers	6		1	1	4
Patient advocates	6	3	1	1	1

The interviews were conducted by telephone and lasted 30–40 minutes. Interview guides were developed in accordance with the research objectives. Among the topics covered in the interview guide were the following: 1) what drove participants to use cloud computing or not, or to consider cloud computing or to decide not to use cloud computing, 2) the ethical/legal/social challenges they faced and how they were addressed and 3) their relationship with cloud service providers (in the case of cloud service providers, with genomic researchers and patient advocates). Three separate interview guides were elaborated whereby the same topics were raised, but formulated in accordance with each group. Finally, while interview data were confronted with documentation (organizational, governmental (regulations/policies), institutional, web sites) in the course of the data collection process, semi-structured interviews constituted the main source of data collection. While the case study approach generally calls for multiple sources of data to be used[25, 26], it can be advanced that interviews conducted with members of different groups constituted a form of triangulation as the information they provided were confronted amongst the different groups of interviewees[27]. Moreover, the decision to opt for interviews as the main source of data collection was a practical one and provided sufficient information to answer the research question[27].

### Data Analysis

Thematic analysis was used to analyze the data using both a deductive and inductive approach [28, 29]. An initial codebook was developed, using Lin & Chen’s diffusion of innovation framework [13] as well as elements of Stieninger et al’s [12] as well as Nedbal et al’s [11] models for analyzing cloud computing adoption. Changes to the codebook were made and additional codes were added in the course of the coding process. Data was coded as it was being transcribed and codes were clustered to constitute potential themes. Themes were generated and developed following a deductive and inductive approach (abductive approach), by confronting elements of the theoretical framework with the empirical data [30–32]. Finally, themes were refined in order to ensure their internal homogeneity and external heterogeneity [19, 28].

## Results

Five major themes were generated to facilitate understanding of how ethical, legal and privacy issues interrelated with considerations regarding the adoption and use of cloud technology among the participants: 1) Getting comfortable with cloud computing; 2) Weighing the advantages and the risks of cloud computing; 3) Reconciling cloud technology with data privacy; 4) Maintaining trust when using cloud technology and 5) Anticipating the cloud by creating the conditions for cloud adoption. These themes aim to highlight the way these stakeholders deal with ethical and legal challenges that shape the adoption and use of cloud computing in their field. Following a brief summary of each theme, themes are presented according to the perspectives of each group along with their specific concerns.

### Getting comfortable with cloud computing

The first theme revolves around the issues, concerns and challenges underlying the process leading to the adoption of cloud technology by genomics researchers. This theme focuses on their main concerns surrounding cloud computing and what renders them comfortable with its adoption. While impressions are similar among the genomics researchers and cloud service providers in that regard, their views differ over the adoption process.

#### Genomics researchers

Considerations over cloud computing adoption were a matter, particularly for researchers, of becoming more comfortable with the technology over time. Cloud computing adoption among researchers depended on the possibility for them to adopt cloud computing at their own pace. A willingness to remain, as close as possible, to a local environment tended to characterize genomics researchers’ preferences:

First, you need to think very clearly about exactly what you want to do and how things are going to work. What we did is that we took all our developers and we gave them time to redesign all of the individual steps of our pipeline such that you can, sort of, metaphorically, click a button and have that particular process go from end to end, totally encapsulated and doesn't need anything else. So for instance, in our data center here, we have a lot of cross-dependencies within our institute for different datasets belonging in different places, getting bits of information in different areas, and of course, when you are in a cloud, you can't do that. We also have steps internally that are automated or semi-automated, and again, in a cloud infrastructure, I would say, it's not impossible, but it's much more difficult to have that human interaction. So we fully automated everything as far as we could and we aslo optimized things as far as we could and that was very very important. Optimization is critical simply because things that run for longer and need more resources cost you a great deal more. So your budget can soon get very quickly (???) by sort of sloppy coding. You need to start from the gound up and think, ok, this is what I want to do, is it the most optimized way of doing it, is it the quickest way of doing it, and then, also, you need to look at the costing models on the other end. Do the costing models match what you've got. So if you use more processors for a shorter period of time, would that cost you less than it would if you had fewer process for a longer period of time. It might surprise what answer you sometimes come back with. (GR0721)

As one researcher illustrated, institutions thus tend to develop ways to keep costs related to data usage down by maintaining what could be called a hybrid setup, that is maintaining a local environment while resorting to the cloud for specific purposes, such as performing analysis:

For our institute, for our own research purposes, I think, we have our own data center, I think that takes care of our immediate issues, but if we need something, then, we go with a private cloud and pay for those extra resources for a short period of time if we needed them. In terms of storage and compute, that's another critical feature that people really need to get their heads around, is how long do you really need that chunk of data on the cloud. The best advice would probably be to get the data up there, which is obviously takes a long time if you are talking about genome data, that can be quite significant for (???). Get the data up there, do whatever analysis you want to get done on it, get the results and then delete it. Close down those vms are whatever else you need up there as quickly as you can, because you're paying for them. The longer they're up there, the longer the data is up there, the more you are paying. (GR0721)

#### Cloud service providers

While, cloud service providers’ account of cloud adoption resembles what was expressed by some researchers, the preferences of researchers to remain in a local environment were surmised by cloud service provides as reflecting a lack of familiarity among researchers with the technology. Thus, cloud adoption in the field of genomics research was described by one cloud service provider as organic, along with the realization among researchers over the cost of maintaining a local infrastructure:

And I think that story plays out for a lot of researchers out there, that they have a problem, they're not sure how to handle some large purchase of equipment that might need answering that problem, and the need to do it either quickly or they don't have the complete funds to invest in large infrastructure or to answer just one question, they can't make that, they have to watch their research dollars very closely. So, we noticed that there was an organic growth in this community, especially in the genomics space, where folks were using not only for the day-to-day research, but also using it as a platform for publishing their algorithms…. (CSP0723)

What emerges, within the cloud service provider community, is the sense that researchers prefer to keep their data partially stored in their local environment, while using cloud computing for certain specific purposes, called a hybrid setup:

I think it feels like it's a bit more kind of a staged adoption of cloud and it doesn't mean that they go wholesale with cloud computing. They may very well live within a hybrid, many of our cases, a hybrid setup is what we see our customers. They have some local resource they've invested in, they have certain applications they are very comfortable running and they've been optimized to run locally. They have some storage that they like to keep some of the data locally and then, they use cloud as a complement where they need to do a scale-out analysis or they have a managed platform that's running in the cloud, or they can use cloud storage as scratch space for the analysis, maybe to even some archival storage using something like [name of storage service] with [name of company]. So I think increasingly, as you described, they may take a step into cloud computing with a partner and then, as they get more comfortable, they decide what's the most effective use of cloud, what's the most effective use of local resources and maybe there's a case to maintain both certainly for some period of time and then, perhaps, over time, they transition more to public cloud if use case suggests that that's the best place to be. (CSP0610)

Lack of familiarity among researchers with the technology and, more specifically, over how to maximize its use, was deemed an inhibitor to cloud computing adoption:

The second concern, which is, I think, now the bigger one, is just learning how. If they want to use a public cloud for genomics, many people don't have training specifically for that. They're just concerned with how they do it. So what are the steps, what are the technologies, they want examples, and so we're seeing that that's a big consideration now, which is the education process. (CSP0612)

One cloud service provider saw its role as one in which it needed to train researchers to use cloud technology. This was further characterized by one cloud service provider as requiring a cultural shift among researchers:

So when we talk about the cloud, in many cases, my experience is that people are thinking about the benefits we get as consumers, but not remembering how hard the culture change was as consumers to start getting those benefits. Because that culture change happened over a long period of time and the cloud showed up and just made it more effective. Whereas in science, all these things are getting served simultaneously, and so some of the biggest barriers to using the cloud won't be technical or legal, they will be cultural on the behalf of the scientists who are not used to a world in which it is cheap and easy to transfer data, it is cheap and easy to replicate experiments and it is increasingly an obligation to submit unused work others can build. All this cloud stuff doesn't work if we don't pay attention to culture. (CSP0609)

Cloud service providers thus see their role as one in which they attempt to simplify the use of cloud technology for genomic researchers. This entails providing a set of services designed to ease the transition towards cloud technology that still enabled researchers to remain in a local environment.

Efforts to respond to researchers’ unease with cloud technology underlie the establishment of what is called a “partner ecosystem” designed to facilitate the transition towards the cloud:

So we, as an organization, we are a very lean organization, and we focus on our core services. So we try to make our compute and storage and networking, our security endpoints as robust as possible, and we concentrate on economies of scale, and because of that singular minded focus, we think we have, essentially, the leading edge platform out there. The flip side of that is that we don't focus on directly helping our customers implement their systems on (name of cloud service provider), but we do have programs and a partner ecosystem, both open source and commercial, that do, just that. So there's a large science ecosystem already present on (name of cloud service provider) that essentially provides pre-configured resources, or platform providers that are specific to genomics or specific to certain problem domains. (CSP0723)

While attributing researchers’ reluctance to adopt cloud technology to a lack of familiarity, as reflected in their preference for a hybrid setup the aim among cloud service providers, becomes that of gearing genomic researchers towards using a public cloud.

#### Patient advocates

Similarly to genomics researchers, patient advocates expressed concerns over difficulty keeping up with cloud technology and the cost related to doing so:

And really, for us, the biggest concern right away was the cost. Two or three years ago, people were very concerned about the cost of storage and the cost of sequencing at that time. As we decided to move forward, a lot of the criticisms that we had to endure were criticisms that you could just, why do this now, knowing you can wait a couple of years and have be a lot cheaper, and I think that underappreciated the urgency that we and our families have to get this done now, but also underappreciated how quickly the price around storage and sequencing has dropped just in the last two years. So we made the right decision. There's a lot of organizations that decided to take the exome route to do a little bit cheaper both on the sequencing side of things, but also on the storage side of things. I think we made the right bet and most people who just did exome are probably wishing they had done whole genome at this point. (PA0729)

But in contrast to genomics researchers, patient advocates do not necessarily remain within the confines of a local environment. Instead, they have undertaken efforts to develop large databases with the aim of facilitating data access for researchers.

### Assessing the cost-effectiveness of cloud technology in the face of security concerns

Decisions over adoption were influenced by its potential advantages were weighed against the risks when considering cloud technology. Such experiences call into question statements over the cost-effectiveness and security of cloud technology in genomics research. Thus, views over cost-effectiveness and security were mixed both within and between groups.

#### Genomics researchers

Cloud computing was deemed by researchers as increasingly necessary to manage and share the vast amounts of genomic data generated following next-generation sequencing. It was recognized that data storage and management is a growing problem and will require cloud technology:

Obviously, the single biggest driver for the cloud, in my opinion, is not the compute, it's the disc storage and in some respects, the data movement. So, in our minds, you pretty much have to have a copy of the data, and that means having enough storage to store what you want. The compute, I think, we can deal with, the disc storage is a bigger problem, and having everyone having multiple copies of very large datasets is what, to me, the cloud really solves. (GR0715)

Cloud technology is deemed cost-effective for its potential to facilitate data management and storage:

So, according to me, the point of using cloud computing is that it makes it economically feasible to handle quantities of data, which you really could not on your single institution system unless your institution has an unusual amount of money. I don't think there is any other very specific example. There is an economic issue. I don't think there's any other argument. (GR0601)

Even when recognizing cloud technology’s potential in terms of cost and data storage, it was mitigated by security concerns among researchers. Researchers remain wary of cloud service providers and seek assurances that the proper security measures are in place. Perceptions regarding the security of cloud technology were thus dependent on these additional precautions:

One thing that I would say is that security is clearly a challenging thing in the world that we live in. There are advantages to the cloud as well as disadvantages and the overall goal has to be to secure data to the greatest extent possible and the cloud is an option that needs to be considered for achieving that objective. (GR0602)

Thus, while the security of the cloud was measured up against the extent to which it was possible to ensure the proper precautions and safeguards, familiarity with how best to proceed to making the necessary adjustments was deemed necessary to ensure that the cloud can be used in a secure fashion:

Yes, many. I think, security is the one that crops up the most significantly and most often. That's not surprising, because to an extent, it certainly feels like you're surrendering control to another individual or to another sort of ethereal body. There are tools provided, but you need to be aware that those tools exist, you need to be very aware what those tools do and don't do, and you need to be aware of what is the most appropriate thing to do for your particular job. For instance, here in our institute, we have numerous friends and colleagues who work in systems support, that take care of many of the systems support-type issues surrounding security and penetration and all that kind of thing. Now, to an extent, in the cloud, those problems are much smaller than you would have in a data center, but we're not systems admin people. We are informaticians, bioinformaticians, computer scientist type people, and so, we need to be aware that there are some times where you need to make sure that you've closed down certain ports or whatever, and those kinds of things need to be borne in mind. (GR0721)

In contrast, such security concerns were characterized by one researcher as an emotional reaction based on the perception of a loss of control over the data:

I don't think this is very complicated, I think it is really quite simple, essentially, the same discussion that takes place whenever some new system comes into place. So the people who are responsible for the data, for reasons which are very clear and valid, have a very high degree of sensitivity to the security of the data. And when you talk about cloud to people who do not have a sophisticated understanding, there is this belief that suddenly, the data are leaving the computer which is sitting in the basement of your building and it's going to some other computer in some other part of the world, and you no longer have control over it. You do not know who can access it, you do not know what they are going to do with it, you do not know how it could be manipulated. And so there is this concern that it would be open to inappropriate use by other agencies and particularly, that you should be outside the legal control of the jurisdiction in which the data are held because it is leaving the geographic confines of the country where the patients or the data subjects are residing. So you have to find a way of reassuring them. (GR0601)

Alongside security concerns were the unanticipated costs associated with installing cloud technology onto already existing IT infrastructure requiring optimization:

So, their concerns were not without basis in the real world, they really weren't. And at one point, I would have given them 50–50 as to whether they were actually correct or not. Making stuff work in an encapsulated form where you run a particular step in the pipeline and to keep going, it runs on its own, that's a massive undertaking to make that happen. The amount of compute time, the amount of resources that were initially required to get this thing to work were truly huge, I mean really, really, really massive. And initially, the suggestion was that it couldn't be done in the cloud, because if you were going to do it, it would cost so much money, it would just be unfeasible, it would be ridiculous. Hence the optimization came in. So our optimization was very lengthy, it took a very long time, months and months and months of development at the time, and it really gave us massive benefits. So initially, they really had a good point in terms of just the resources and the requirements were huge, and of course, going into the cloud infrastructure, you lose, because of your extra overheads or your various hyper-visors and layers and obfuscation and whatever, I mean, you've got probably twenty percent overhead. So you probably, you're getting much less bang for your buck as it were. So these were reasonable concerns to have at the outset. (GR0721)

Concerns over the variable cost of data usage, which came as a surprise to some researchers, were also expressed. The difficulty lies in keeping track of the use of cloud resources at peak times and ensuring that they are optimizing their cloud usage during non-peak hours:

We didn't anticipate just how much the cost fluctuates. I cannot stress that enough. Well worth looking at the graphs that are available, if (name of cloud service provider) or (name of cloud service provider) do make them available, but honestly, it's really quite surprising how much it fluctuates. We did not really fully comprehend how long it was going to take the data up there. We did not fully really appreciate just how much compute time we were going to need in a cloud-based environment. We underestimated basically. And I think these are very important and useful lessons. It's fair to say that nothing on this scale has ever been done before, and I think we all have. (GR0721)

Ongoing fears over the security of cloud computing and the addition of monitoring options shaped researchers’ views over the technology’s cost-effectiveness. While a sense of a loss of control over the data prevails among researchers as they undertake additional security measures in order to protect the data despite providers’ security reassurances, a balance has thus yet to be achieved between cost-effectiveness, security and data storage:

I think it's all about economics. Who can do this most efficiently while still protecting the security of the data and the privacy of the individuals who donated the data. That's the key. (GR0731)

#### Cloud service providers

Cloud service providers tend to defend cloud technology for its security and argue that it is as secure, if not more so, than other already existing technologies used to store and analyze genomic data:

For example, because no data are in the computer. Many times, people, for example, lose a laptop or a laptop is stolen or a USB storage device. So you have the security measures in the cloud. The cloud is outside of those devices. It's more secure. Then, if you have the recognizable security measures in the cloud, the cloud is outside of any of those devices. It's more secure in that sense. (CSP0522)

Cloud service providers tended to attribute precautions taken by some researchers as reflective of an emotional, or irrational concern, regarding the security of the cloud:

Well, customers, in general, they don't mind how we do the analysis in general. But cloud computing, in the end, for them, is more scalable, cheaper and, in my opinion, more secure. But some of them, I think that a minority, they have some concern about cloud, but not scientifically-based concerns. It's more an emotional concern. Emotional in terms of that they have heard that the cloud is not secure, but this is not the case. This is not indeed the case. (CSP0522)

#### Patient advocates

Similar to cloud service providers, there was a tendency within the patient advocacy community to put forth the security of cloud technology by comparison to other technologies:

For one thing, the data don't reside in one place, there's expandability, the elasticity of a cloud environment allows it to expand as your data needs expanding. So there's less likelihood that you're going to overload the server for example. Something that happens fairly often in a data center, if the equipment isn't sized correctly and as far as backup and failover, cloud environments are built to do, switchover and failover to a backup server if a component goes bad. I mean that's what they're built; they're built to be highly highly available. And so from availability is a security concern. As I said, security deals with confidentiality, the integrity of data and the availability of data and services. So the availability metrics for cloud computing are far better than they are for data centers. (PA0604)

Not only is the cloud deemed secure, but the potential for the technology to keep better track of who is using the data and for what purposes was pointed out as underlying the security of the cloud:

We could track how people, using the data in the way that they've been approved and the same computer environment makes the usage possible, the access possible, can also police or control who's using what data and under what circumstances. And so it, part of the solution, part of the problem is that it creates much easier access to much larger sets of data and also creates the mechanisms by which you control it at a fairly granular level. Who sees what kind of data and if somebody tries to see data that they are not authorized to see, the machine can identify that and create the exceptions. So, a lot of this is about access and identify management, audit trails. The technology is there to do the control, but the human dimension is essentially the same as it was in a pre-cloud environment. You want honest people doing honest things and being above board in what they want to do and why they want to do it, and if you say you've got permission to access data, then, you have permission or consent. So the human behavior is the same, and if we get this right, the systems designed to increase access will at the same time create the tools and the mechanisms to control access and to deal with data breaches. (PA0512)

With respect to security, researchers are more cautious while cloud service providers and patient advocates are assured regarding cloud technology safety. This is in spite of researchers recognizing the need to use cloud technology to facilitate the storage and management of genomic data.

### Reconciling data sharing and data privacy

Underlying decisions to adopt cloud computing are notions of data privacy that are embedded in various policies and regulations, be they jurisdictional or institutional. The challenge then lies in reconciling the technology with data protection provisions reminiscent of a pre-cloud context. The development of alternative consent mechanisms designed to work within the technology emerges as a possible vehicle through which to ensure data privacy in a cloud environment. A major concern with regards to data privacy and consent relates to the anonymization of genomics data and its repercussions in terms of data privacy. As genomics researchers, patient advocates and cloud service providers share these concerns of increasing data sharing while ensuring data privacy, the redefinition of consent mechanisms as a response to the conundrum over data sharing and data privacy is met with divergent approaches among genomics researchers, cloud service providers and patient advocates.

#### Genomics researchers

Interest in data sharing among genomics researchers appears mitigated. While there is much eagerness that is expressed over the need to share data, cloud technology as a means to put forth data sharing was not deemed a priority nor part of their work, as the relevance of data sharing itself was put into question by one researcher:

In terms of the benefits, it seems that, in my opinion, for at least some groups and some things, I think the benefits are quite exaggerated. What we really need for medical use, making a medical findings, is control of everything. So it's not attractive to us to use a pipeline that somebody else has set up because my assessment of the level of expertise of a lot of the commercial companies is that it's not really state of the art. (GR0520)

One major characteristic of the research environment that hinders data sharing is the tendency by researchers and academic institutions to restrict access researcher and academic institution databases in an attempt to secure their data for publication and/or patent purposes:

Well, their concern is that if they let it out of their institution, somebody's going to make it public or release it in some way that will violate the patient's privacy. So they feel they owe it to the patient to keep their data locked up. They cite their own compliance rules and regulations and their boards and so forth that have told them that. So there's a history in biomedicine for, if it's a research facility, for the researchers to keep their data to themselves for as long as possible so they can keep publishing information on it, making discoveries that they don't want anybody to steal their data and make discoveries before they get a chance to. So all of those things come into play. (GR0731)

The result is the siloing of databases, thus inhibiting broad data sharing as it involves restricting access to institutional members:

You have to have access to other genomes and right now, most of the medical institutes around the world have policies to force them to silo their information away. So every, all the genomes that are sequenced in their institute are kept in their own database and no one outside of that institute has access to that. It will never get anywhere with medicine if we continue this way. (GR0731)

While some researchers recognize the need for some restriction, they select collaborators based on their willingness to engage in data sharing. At the same time, they recognize that the data may have more than just intrinsic value:

So the big challenges there are, it really centers around how data are used, is it anyone can do anything with any data, or is it that I license you to use my data in some way x, y, z, and I approve of those. And so, I would say that within our institution, we have a range of opinions on what the right platform is, and I think the aspiration would be that data, once data are in the network, they can be used for any good reason, but the recognition that that might not always be possible. So we're trying to identify partners who are most sympathetic to the view that data should be shared broadly. But, I think the challenge is to sort of deal with the idea that some people may want to restrict access to their data for certain individuals for remuneration or collaboration or whatever reason. That, I think, is one big challenge, that data have value and you have to decide on how that value is shared. Is it given away for free or is there some exchange of other value for it. (GR0715)

Thus, calls for increased data sharing are confronted by an academic culture that prioritizes retention of data for publication and/or patenting purposes and cite privacy and security concerns as a means to do so and as a basis to restrict access to data and thus inhibit data sharing.

Alongside researchers’ reluctance towards data sharing, is a tendency to shy away from privacy issues or, in the least, those issues that are specific to the use of genomic data. Thus, reliance on traditional consent mechanisms persists as it is deemed sufficient to ensure privacy (GR0602). The sense among researchers is that cloud computing does not change their obligation to secure ethical approval to share data in terms of privacy. On the one hand, there is the notion that, from an ethical standpoint, the problems facing cloud computing are the same as in the absence of cloud technology, in that researchers must still obtain ethical approval from various institutions in order to share their data with one another:

The ethical side of things is another interesting one. I mean what, how do you decide, so if you are going to follow my model of, you've got data in a cloud and you meet in a cloud to do some analysis on a particular dataset, whatever that analysis might be, have you got all the appropriate ethical approval to do that work. So you could argue that that's a concern that may be, that's not a huge issue because we would have to consider that anyway. So if we were going to do the work here, on site, here in (name of city), or if we were going to do the work at some remote cloud infrastructure, that's similar, if not identical. We would have to have appropriate ethical approval to do either. I don't know how people would generally, people in the broader sense, be that grant awarding bodies or patient advocates or patients themselves, how they would feel about their data being processed or worked on in a cloud infrastructure versus a data center like ours, I don't know. (GR0721)

Alternately, cloud computing is thought to be no different in terms of privacy than using a credit card. Just as one consents to having their credit card company have access to their personal information, a similar situation was thought to characterize the use of genomic data by researchers:

When you actually had to sign the form and they'd look at the signature, they say ok, it looks like your signature, ok, we'll let you have this product. Now, you just go in there and tap with your card, which could take off the street, a hundred bucks is taken out of your account like that, which you are liable for by the way. If you lose your card with that near-field communication thing on it, if you lose your card, through no fault of your own, and it goes tap tap in five different stores in half and hour, each hundred bucks which you are liable for. Because it's easier, we've agreed to it. So similarly, we've agreed to things on the cloud, or whatever, because it's easier, but there is a price to pay for it. (GR0619)

Privacy becomes perceived as a price to pay for facilitated access to data. This loss of privacy runs the risk of going unnoticed with cloud technology adoption:

Predicting the future is always dangerous right. I mean you could almost see a time whereby, there's so much of our data in the cloud right now, Facebook, EBay, Amazon, all of that kind of stuff, there's probably got far more identifiable data than any of the rest and it's kind of interesting to think that they are and aren't doing with it. But you could take one perspective and sit back and think, well, you know, in the future, you can see where people don't really care about cloud stuff, it's just that they have access to the data wherever they are. It would actually be kind of useful if you could go to any doctor surgery in the (name of country) or in the world and be able for that particular GP or consultant or whatever to have access to your medical records straight away, wherever they are, and the only way to do that would be in some sort of cloud-based system. So, it's kind of a tricky one to think about. I think we're crossing a boundary here. But I think there's a great force of good, potential in the offing. (GR0721)

One element that raises questions regarding the sufficiency of already current consent mechanisms to ensure data privacy is the risk that one’s genomic data may be identifiable and, thus, the possibility for the privacy of genomic data to be protected:

So the risk of re-identification, that I already have your sequence and now, I want to see, did you have cancer, and what was your treatment and all that stuff, that I can do no problem. And that's been understood for quite some time. And that is why (name of database) protects its information. But what is not so understood is whether or not the DNA sequence itself, all by itself, is identifiable, without having access to the DNA sequence of that individual. (GR0715)

However, views differ in that regard among researchers interviewed. One researcher was of the view that only if genomic data is combined with other sources of information can it be used to identify an individual:

If it's a research environment in particular, you can go to considerable lengths to make sure that your data are anonymized. You can go to considerable lengths to code data as they are transmitted. That is what they do, I mean, that is what all the financial institutions do, it's what all commercial organizations do. And it's not perfect. The question is whether it's good enough. It's not perfect, but it's certainly is not a system which in biomedical research, has given rise to concern, or at least, which is given rise to abuse. There are very very few instances of biomedical research data being abused in any way apart from an occasional academic misbehavior, like people publishing a paper before someone else. So that's naughty because they said they wouldn't, but it isn't really an invasion of the data subject's privacy. It's an invasion of the rights of the researcher. (GR0601)

Others rather maintain that genomic data alone can identify an individual and thus requires that privacy issues be taken seriously and go beyond the usual safeguards, which do more to inhibit data sharing than to ensure privacy of genomic data:

These were whole genome data. So every read in the file produced in the sequencing machine. So these kinds of data bring up privacy issues, even if there is no name associated with them and no specific identifiable information, like social security numbers or addresses or something. Just by the nature of them being DNA and being in a repository of cancer genome, that brings up privacy issues that we deeply respect. (GR0731)

While some questioned the extent to which patients actually care about how their genomic data is used (GR0715; GR0721), they still recognized the possibility for changes to be made to the consent process, particularly given that genomic data could be used for different research purposes over time:

For me, I think we need to inspire confidence and I think we need to listen to people, but I think that discussions around consent and particularly informed consent rather than just consent ticking a box on a piece of paper when you don't really know what it means. I think that's probably the hardest and how do you explain to somebody what it is that's going to happen to them, because in some cases, it's very complicated. I don't know the answer to that, that's a very tricky one. (GR0721)

However, questions remain regarding the extent to which it was possible for patients to keep track of how their data was used over time:

It is absolutely true that the data will be used over time. I don't think the accounting step of saying that people have a right to know who is using their data and for what purposes is possible to do. I think that that level is, I mean, in a cloud environment, you actually could keep track of what was done, what algorithms were run and who ran them and all that sort of stuff. So there is some advantage to that, but whether or not it makes sense to do that, I actually don't think it does, but keeping such a log is valuable, we would actually keep such a log on our private cloud computing infrastructure. And we would do that to work to ensure privacy, to make sure someone wasn't trying to gain access to the system (GR0715)

Thus, genomics researchers are struggling with how best to facilitate data sharing and ensure data privacy, while adhering to academic notions of data privacy, data protection and consent that may not fully address the complexity of protecting the privacy of patients’ genomic data. Such issues, may ultimately lead researchers to withhold their data.

#### Cloud service providers

According to cloud service providers, a lack of consideration tends to characterize the research environment with regards to privacy issues. Their perception of researchers is that of viewing privacy as a hindrance to pursuing their research:

…In general, my perception is that people in the biological research area don't particularly want to become domain experts on security and privacy and policy management. They'd either like to ignore the problem or to have those problems taken care of in a way that isn't disruptive to their work…. Long-term, I don't think that that's a realistic attitude, because I believe that he, like many other people, is working in an environment where these data belong to actual people who have legitimate privacy interests in the data, and people have to be cognizant of that fact when they are working with the data and I believe that means that they'll have to be subject to some constraints on the use of the data. It can't be complete carte blanche with this data. I think there is that attitude. I think there's an attitude that privacy comes at the expense of access and utility. So, in other words, if you want more access and you want more utility, you have to have less privacy and if you want more privacy, etc. So there's some kind of trade-off. I don't believe that that's completely true. In fact, we're endeavoring to show that that isn't true with (name of cloud service provider). But what I believe is that there are pragmatic ways to balance the privacy of the data with access. (CSP0623)

Cloud service providers also perceived differences between genomics researchers and patient advocates with regards to privacy issues, as genomics researchers tend to be more wary than patient advocates, who tend to prioritize facilitating data sharing as a means to further the development of treatments:

Interestingly, in some ways, their concerns are almost diametrically opposed to what researchers are worried about. Researchers and public health professionals tend to be very wary about the risks of accidentally disclosing information and they tend to be very restrictive about how they allow data access. For example, they may have a policy in place where data is only consented for specific uses, researchers have to apply for access, they have to go through an application process, submit a research use statement and be approved. Then, that approval is only for that particular research use for a particular bounded period of time. What we found with many of these patient advocacy groups is that they are concerned about and they believe, many patients believe that sharing their data as widely as possible in the least restrictive way they can will help lead to insight that can help people like them. So often, we find there's pressure from advocacy groups to be less restrictive, to make data more available, to have more open patient consent, less onerous data access request policies and in some cases, make data available not only to researchers, but to citizen-scientists and other patients. So there's pressure to make things more open. And that raises interesting ethical issues. (CSP0612)

As the patient advocacy community’s stance on privacy issues and data sharing converge with those of cloud service providers, a definition of privacy in which the patient can control how their data is used and by whom is being put forth as a basis among certain cloud service providers for developing alternative consent mechanisms that would place patients at the center of the decision-making process surrounding data access and sharing:

So in the current world, in (name of country), for instance, you donate your data, some research ethics board approves the study and allows that researcher to use data. And in (name of state/province), they have special repositories of information under (name of government body) that can collect information and give it to researchers. So there's a regulatory regime in place. So you sort of rely on the regulatory regime to protect you. You don't really have much visibility in what's happening, you rely on the fact that there's some laws in place and hopefully, someone is willing to enforce them. And it's an open question as to whether or not that is sufficient. It may be good, but the question is does it provide privacy protection? Because privacy is not about letting a government official decide what to do with your data, because the Stasi in East Germany could do that. Privacy is about you deciding what to do with your data. (CSP0527)

Alongside increased patient control over their data is the notion of data ownership as pertaining to different stakeholders:

This is a slight editorializing on my part, but I think that the idea of ownership of healthcare data is a limiting idea. When we think about it terms of who owns the data, I think that that limits our understanding of the reality. It's better to think about data being governed by a series of stakeholders, all of whom have some interest in how it is used. So in a clinical setting, just to take an example, you have a patient, you have a physician, the physician works for a hospital, the hospital is possibly owned by some larger corporation that's in a state, in a country. So there are multiple, there are national level interests in terms of epidemiology for tracing disease and things like that. So there are a multitude of stakeholders who have some ethical interest, some right to modulate how the data are used and I think that the healthcare IT systems that are out there today tend to ignore the idea that there are multiple stakeholders in each and every dataset. They tend to look at who holds it, who is physically in possession of the data. So again the attitude of possession is nine tenths of the law. I think it's a limiting view. (CSP0623)

Cloud service providers in response to data sharing and privacy challenges have developed alternative consent mechanisms that seek to challenge existing ideas of data ownership such that it increases patients’ control over their data, with the goal of increased data sharing and access.

#### Patient advocates

While there is strong recognition within the patient advocacy community for the need to increase data sharing, doubts are raised within that community as to the extent to which data sharing is occurring, which was attributed to ongoing and unresolved privacy issues:

More sharing, they patients want to see it shared. For rare diseases, and cancer, of course, is increasingly becoming a rare disease, ….It has huge benefits and it allows global sharing of DNA or global sharing of sequences, which is particularly important for rare diseases, and people like in the pediatrics community. So patients get very engaged in that and organizations that do, like rare genetic disease organizations etc, would be very informed about all this kind of thing, but I think it's only small pockets like that, where people really see the value of their particular disease where they understand all the implications from the privacy and the ethics and all that. People think that they're sharing and walking away. (PA0303)

Similarly to some genomics researchers, one patient advocate pointed to academia as impeding data sharing:

What we have been troubled with is when it comes to large, particularly genetics data, we live in a world of privileged access. Large-scale genetic datasets have really been owned, if you will, by extremely well-funded academic labs that are based in centers with large compute facilities that can actually support the kind of analyses that ever-evolving technology around genomics is making available and what has always bothered us is that these labs sit on the data for extended periods of time and there's not rapid immediate release of data to help support innovation that is available out there in the exploitation and the analysis of that data that could come if the data were available more openly. And usually, you've got to wait for extended periods of time until the labs have squeezed every drop of value from a publication point of view, even a patent point of view out of that data before they will release it and, even after they agree to do that, there are some challenges in making data more broadly available to the larger community. (PA0729)

Certain initiatives within the patient advocacy community are thus developed with the aim to facilitate data sharing for researchers by providing them with a database outside of academia:

We definitely wanted to disrupt that privileged access model and create a level-playing field for innovation. And by doing so, we knew that we are going to fund our own work rather than depend on grants from the governments, etc. We funded our own work, we would have a lot more control over the policies around how that data was made available. If we were the ones funding the sequencing of our samples and we didn't feel an obligation to analyze and publish on that in order for our return on investment to be met. We could make that data available right away, which is exactly the decision we made to do. So that's itself disruptive, to have a single force driving the sequencing of samples and driving the generation of data, or creation of value in a way that doesn't expect the institutions doing that to get a return on investment by controlling the access. And so, by doing that, we were able to create database and decide how and when we'd like to make that data available and our general inclination or our thesis, if you will, behind (name of database), as the more eyes that we can get on a dataset like that, 10 000 genomes from multiplex families with autism, the more eyes that we can get on that data, the greater the probability of discoveries and innovations the exploitation of that data would be made. (PA0729)

The development of alternative consent mechanisms is also put forth by patient advocates as a means to navigate siloed databases without genomics researchers having to address policies and regulations directly, which is considered a lengthy process:

…the issue is that if I am a patient, my data actually exists in multiple institutional silos and those silos, each have an obligation for security to keep the data protected, but none of them have, they're in effect like columns in a great big table, whereas the patients, the individuals to whom those data pertain are like the rows in the columns. And so, what I want is to have the individual be able to control through access rights, the ability to de-silo the data. (PA0622)

Researchers’ reluctance to share data is also viewed as an opportunity for further collaboration between patient advocates and cloud service providers whose preferences for data sharing and data access coalesce:

And you're not going to find that in a lot of academic institutions that are looking to monetize the creation of resources like this or companies that are much more concerned about the value of data and want to limit access to it. So I think you can see where a nonprofit can slide into a collaboration with a (name of cloud service provider)-like organization quickly in a space that is filled with a lot of folks that are motivated differently. (PA0729)

The risk that access to genomic data may, in and of itself, identify a patient, also underlie efforts within the patient advocacy community to provide patients with increased control over their data. Some patients’ strong willingness to share and donate their data for research purposes provides the impetus behind developing alternative consent mechanisms in a way that is minimally disruptive to their lives:

But when you're asking for whole genome sharing on a broad sharing basis, without where it goes into the cloud, and you call it de-identified information because you take the name off of it, and you know darn well that it's re-identifiable because it's a biometrics. I don't think people are, I think there's a desire for sharing, I know why it's occurring…. I know why researchers need access to data desperately and I know that patients are as eager as anyone in the equation. They're much more eager than pharmaceutical companies and they're much more eager than researchers to have their data result in breakthroughs. They're the ones suffering. So I know that the eagerness is there from the patient perspective, but I don't believe there's an empowerment that we're giving those individuals to express that and I know, I can tell you stories … I've gone to national conferences where I've encountered other people with the same condition and had young men say to me I could never to what you've done because I want to be in the US military and if I disclose that I have the condition, I would be eliminated from military service. And they're able-bodied capable individuals and they want to be in the military. So they're prepared to not disclose that they have the condition and to practice privacy by lying and omission in order to lead their life in the way they wish to lead it. That's a lousy way to accomplish things. (PA0622)

Efforts to align consent mechanisms with cloud technology engender a redefinition of data privacy that comprises both increased patient control and multi-stakeholder data ownership. With increased patient control comes the possibility to filter through the different institutional and jurisdictional silos within which their data can be found. It becomes incumbent upon the patient to navigate these silos as a way of providing consent. Finally, the difficulty of protecting genomic data through encryption or anonymization, paved the way for patient advocacy organizations with the opportunity to develop alternative consent mechanisms that reflect the aim of facilitating data sharing through cloud computing adoption. Such aims converge with those of cloud service providers who are rethinking traditional notions of data ownership.

### Maintaining trust in the cloud

Accountability issues specific to the cloud arise from the difficulty to assess the source of a breach, regardless of whether a breach is due to human error or malicious intent. Establishing who is responsible in the event of a breach becomes all the more difficult as data may be stored in data centers situated in different jurisdictions and may be transferred from one jurisdiction to another without the researcher being able to keep track of their data. Cloud service providers store the data in data centers in different jurisdictions in order to ensure the flexibility and the speed of the technology and, in the process, take it upon themselves to navigate between these jurisdictions. How exactly they go about doing so, however, remains unclear. Different perspectives exist of how trust is maintained in the cloud in the face of jurisdictional differences and a fluid cloud environment that renders it difficult to pinpoint the location of the data and, thus, the origin of a breach.

#### Genomics researchers

Cloud computing adoption rests on the extent to which trust characterizes the relationship between cloud service providers as well as genomics researchers. The situation is especially critical for researchers who are held legally responsible for the data stored in the cloud. The need for them to do so stems in large part from the need for them to ensure that the data that is stored be deemed compliant with regulations aimed at ensuring the privacy of patient data, such as the Health Insurance Portability and Accountability Act (HIPAA) in the US. Under HIPAA, it falls upon the data holder, or the covered entity under HIPAA, to ensure that the data stored onto the cloud is de-identified. But while the data that is stored onto the cloud may be transferred between different jurisdictions, it then becomes difficult for data holders, in particular researchers, to keep track of. As a result, the data holder cannot directly know how the data that they have stored is being manipulated. As data transfer and jurisdictional issues complicate the relationship between cloud service providers and researchers, a reluctance to adopt a public cloud ensues. Instead, the tendency is to opt for a private cloud, as it is thought that the risk of a breach of the data is lower:

We are not envisioning moving the data out of local sites. So it's not a cloud environment in the sense that you don't know where your data are. It is a distributed computer environment and you can compute on data that exists remotely without, and you may not necessarily be able to access those data, but it is not one in which the data are distributed in a way that you don't understand. (GR0715)

As put forth by one researcher, data control was managed through utilizing a variety of tools:

I think it's a combination of the contract, the auditing mechanisms put in place and understanding the networked typology and the whole way the vendor system is constructed so that we understand what risks take place. (GR0602)

One researcher, however, considered such jurisdictional preoccupations as no different than those related to ensuring the safekeeping of the data and to human error:

My background, I have much more experience with hospitals than I do with big research institutions. So the great majority of breaches of patient privacy and confidentiality that I never got have nothing to do with research and nothing to do with clouds and actually, often, not much to do with computers. They have to do with people who take some patient records home because they want to do some work at night and they leave them lying on the train or something like that. They lose things, they are human beings. That's where most of the trouble comes from. So all of this extreme anxiety about computing ignores the fact that most of the trouble doesn't come from computers, it actually comes from human error. (GR0601)

An alternate jurisdictional strategy is simply to prevent the data from being stored in other jurisdictions outside the institutions:

That's something we prevent from happening. Transfer form one jurisdiction to another, unless it's part of a research program in which case, in our view, what they're really doing is consenting to the research program, they need to understand what data movement is occurring as a result them being in that research program or in whatever program they are a part of. So we just view it as one level up, that it's not something that we will make sure that the cloud service that we use comply with whatever the patient is consenting for and we need them to understand the highest level what the risks and benefits and the cloud is just one part of that. (GR0602)

Finally, the absence of a legal framework designed to sanction breaches and frame uses that occur outside of their jurisdiction adds to researchers’ fears over the loss of control over their data:

Yeah, I think the ethical thing is an interesting one because I think that's going to come down to national boundaries and think it's going to take time for legislation to really catch up and for people to really get comfortable with that. In terms of the physical, what sort of sign off you need to use data, you should have that authority, you should have that ethical approval to use that data, whether it's in your own data center or somewhere else, and that shouldn't really be hugely different, as long as the people who are giving the data are happy with it being cloud-based. (GR0721)

In the absence of such a framework, a sense of powerlessness in the face of cloud technology transpires, as expressed by one researcher:

I guess you have to trust people. You just hope that they are not abusing it. I don't have much control over what they do with their data. I kind of assume that they're, we send people's names. Obviously, you could work out who these people were if you wanted, as you probably know. This can be done by all sorts of methods and analyzing the sequence, you can know who somebody is, if their sequence is available in other places. (GR0619)

#### Cloud service providers

Cloud service providers tend to underline that it falls upon the researchers to ensure that the data stored in the cloud is secure and to decide, in line with current privacy regulations (HIPAA in the US), what constitutes personal health information:

We talk about that all the time and it really boils down to [name of company] doesn't view data, it really is the customer's responsibility to secure the data and make sure that if they are handling personal health information, which increasingly, genomic is looking like it's going to be classified as, then, they need to protect it in the same manner than they would other personal health information on our platform and it falls under the HIPAA program, a [business associates agreement] BAA program or the equivalent of such a program within other regions. (CSP0723)

The challenge for researchers, according to cloud service providers, then lies in the possibility to monitor and control where the data is stored as well as transferred, which involves not only contractual agreements, but also the possibility to audit their cloud service provider:

I'd say a deeper problem than that is the ability of the customer to audit security practices of the cloud service provider. It's one thing to have it in the contract, that's nice, but a contract is only the record of an agreement. So the next step is how can you be sure that your cloud service provider, whoever it is, is actually living up to their obligations. That's a deeper problem where you get into things like (???) schemes and audits and all the rest. (CSP0527)

Various combinations of auditing practices were highlighted as ranging from cloud service providers developing audit-logging mechanisms that enable customers to keep track of their data, to genomics researchers hiring an external agency. At the same time, it was also noted that genomics researchers’ ability to do so, according to one cloud service provider, rests on the resources and expertise they have at their disposal to verify that their cloud service provider lives up to its contractual obligations, especially if these include geographical restrictions:

Yeah, and this is an issue, right. It's not easy, you're not going to take a Ph.D. in biology and send him into a data center. They need some expertise …There are ways to do it certainly and this is why it's not that hard of a problem. I'm not saying it's easy, but I've seen mechanisms where this can be done. So an organization like [name of hospital], they hire some expertise there, they hired people from Silicon Valley. They certainly could go in and talk to [name of company] about their current cloud practices and [name of company] could give them their list of latest security audits and their list of controls and they can have a discussion about it. (CSP0527)

Furthermore, one cloud service provider pointed out that contractual provisions may not necessarily provide clarity as to who is liable in the event of a breach, since it remains difficult to ascertain whether a breach affects the integrity of the data:

It's a little bit murky though because at the end of the day, the covered entity, under the law, the one who collected the data and who is storing it, who sets all of that into motion, that person is the covered entity who's liable for privacy. So it's incumbent upon them to make sure that the third party providers that they are using appropriate legal agreements that deal with who's liable in the case of a breach or they have enough faith in the architecture and the design of their system to feel like the risk is acceptable. It's actually quite a complicated question as to who is liable. (CSP0623)

Moreover, according to one interviewee, the customer (the genomics researcher or patient advocate) is most likely to be deemed liable in the event of a breach:

Well, that has to be taken on a case-by-case basis. Because if it's a breach from our API endpoints, things we have determined are within the eligible services of that business associates agreement, then, we would be liable for breaches. But the way the program is structured, that risk profile of actually (name of company) being the source of the breach is much smaller than the customer-level responsibility. Because again, they are responsible for all the applications and operating systems that are in the cloud. (CSP0723)

While researchers are challenged by their legal obligations, data privacy and control concerns, cloud service providers nevertheless attempt to act in accordance with the demands made by their customers. To meet these demands and ensure that the technology performs, data is stored in different data centers or jurisdictional zones to ensure the flexibility and efficiency of cloud technology. Two large cloud service infrastructure providers (that were part of this study) do so by delimiting data centers across regions (US, EU, Asia) that allow a customer to restrict their data storage within one jurisdiction:

Exactly … It's different for every service because some of our services are whole region services. So we have this notion of a region-within-region system for data centers. But the reason it works and the reason it allows, it looks for data corruption and repairs it, is because it's already copied data to three different data centers. Therefore, it's spread across the region. It's still within the region but it's spread across the region. But something like our devices, networked attached storage devices called EBS, they are going to be specific to a data center because they are going to be attached to a running server. So for EBS, it's down to the data center. And the customer decides where to move it around. So it's different for the services, but the gist is that the largest hub for any data within (name of cloud service provider) is a region. And that's geographical. (CSP0723)

From the perspective of one the cloud service providers, such a setup also provides them with the possibility to store the data in different data centers in case of a breach or system failure, while respecting their customer’s wishes:

There's two aspects of that. There's technology to control data locality and limit where data can move and then, there's policy, which is the other way of handling that. If you make your user sign an agreement saying that they want to move data outside of your jurisdiction, and into certain jurisdictions, then, you have a legal contract to enforce even if it's not technically enforced. Technologically, as a cloud service provider, we do have settings that you can apply to your data storage for where the data will be stored at rest. For example, if you want your data to never enter the US, you could choose data localities for example in the EU and you could restrict your data to only reside within the EU. Or if you want your data to never leave the US, you could configure your data locality to be stored only within the US national borders. (CSP0612)

Apart from mechanisms aimed at ensuring audit control, other strategies were put forth by cloud service providers that leaned towards developing common approaches as a way of ensuring accountability. Creating federated data networks as a means to circumvent jurisdictional issues constituted one such strategy:

In fact, I would argue that if we really want to capitalize on the potential of genomic data, that tends to mean that we need to federate. Because the really interesting variants are rare, the things that will kill you are, by evolution, they don't occur that often. So what I have learned from working in this area for the last couple of years is that there's a very strong imperative for people to federate datasets and for researchers to be able to access datasets across the planet to build more and more statistical power and better and better sample sets. If we're going to do that, if that's the direction that the field needs to go, in the direction of federating more and more datasets, then we must address the issues of privacy and security of data head-on. (CSP0623)

The tendency, within the cloud service provider community, is to attribute much of the legal responsibility to researchers, even as they adhere to efforts aimed at developing common approaches and practices. Further, offers to provide researchers with the option to decide where to store their data are dependent on researchers having the resources and the expertise to negotiate their contractual agreement in accordance with their security concerns.

#### Patient advocates

While the perspective within the patient advocacy community is that the onus is on the researcher and their institution when selecting a cloud service provider (PA0604), one patient advocate maintained that it was not possible to change settings or the location of the data being stored, particularly when it comes to large databases:

Because of the impossibility of making that happen. It would be an exception to how (name of cloud service provider) operates to insist on petabytes, if we were insisting that portions of that dataset or the entire portion of our database that we are building be maintained in a partition that only exists within (name of cloud service provider) or a list of countries, what the technical requirements of fulfilling that would be, I think, enough to dissuade them from wanting to have us as a customer. (PA0729)

At the same time, the relationship between one patient advocacy organization and their cloud service provider was viewed as being aligned along a similar vision:

I think the relationship emerged independent of genomics, for reasons that I am describing. We have a lot of alignment with (name of cloud service provider) on a variety of different issues that affect the autism community. Their business in genomics was evolving at the right time that our other conversations started. But we were very quickly, as we started to explore how we could work together, a non-profit foundation working in the autism field with (name of cloud service provider) who desires to work with philanthropic organizations on different fronts, as we started to explore that, we were very quick to identify this as a perfect opportunity for us to collaborate. Here's a non-profit with an unprecedented sample set and wanting to build a cloud-based database and make it available to the world, talking to a company that wanted to leverage all of their assets to build a genomics business to make genomics data available to the world. It was perfect timing. So, we acted on that very quickly and we're both relatively young organizations, we think very big, we're both very inclined to try to take on problems of great human significance (PA0729)

In that setting, means are being developed within the patient advocacy community, in collaboration with their cloud service provider, to facilitate access to large amounts of data and represent a means of circumventing the dilemma in which researchers are at once solely responsible for the data stored onto the cloud, yet are limited in their possibility to keep track of that data and, in the process, their cloud service provider. Among such strategies is one whereby the data remains in the organization in question and cannot be downloaded by researchers:

I think it's a very real issue and it's a reflection in a sense of the relative ease with which you can collect data and the extreme difficulty with which you can monitor what is done with it, particularly if you allow the export of data from your own, as it were, safe haven. The approach that (name of company) has taken for example, is the model of the reading library rather than the lending library. They will have sensitive genomic and health related information about a hundred thousand (name of a country) health service patients which will need to be interrogated by academics, by industry or whatever. But whatever they've said is you can look at our data in our facility, but you can't export our data for your own purposes to another place, and if anyone is found doing that, then, essentially, they will be preventing absolutely from darkening the door of (name of company) ever again under any circumstances …And you can obviously consult it if you fulfill certain requirements, if you are a bona fide researcher, the project you want has got ethical approval, all that sort of thing …you have to go through a set of validation procedures. (PA0616)

Another one those means include providing incentives towards preventing abuses by sanctioning those that use the data that is stored for purposes other than research:

…the need for encouraging and supporting good behavior. This will be a recurring theme, that you're more likely to get people, if you give them the tools, give them the encouragement, give them the reinforcement for doing the right things, it's likely to have a greater impact than sanctions to people doing things wrong. So if we can make sure that people know what they should be doing and there are able to do what they should be doing, that there'll be less of a need for sanctions or expulsion for people who don't do it because we'll just reduce it (?) and because most of the breaches are likely to be through ignorance or accident, rather than malfeasance. (PA0512)

Divergences persist between groups regarding the extent to which the data owner may have control over the data that is stored onto the cloud, even as the responsibility remains in the hands of the data owner in the event of a breach. Cloud service providers contend that the capacity for researchers to monitor and control how and where cloud service providers store their data depends on their negotiating capacity and resources to audit the cloud service provider. Patient advocates, for their part, offer alternatives to researchers as their database are made available for consultation, as their relationship with cloud service providers, particularly compared to genomics researchers, is closer. Nevertheless, similarly to genomics researchers, they too remain skeptical regarding the possibility to monitor how cloud service providers store their data.

### Anticipating the cloud by creating the conditions for cloud adoption

Part of what influences cloud computing adoption stems from efforts to normalize its use in genomics research, in an effort to increase public trust in the technology. As public perceptions over cloud technology as well as data privacy tend to shape policies and regulations, the development of standards seek to provide a means of navigating around such policies and regulations, which are not deemed adapted for the purposes of genomics research. Still, concerns over public trust, particularly among genomics researchers, persist and thus shape efforts in anticipation of a cloud environment.

#### Genomics researchers

The inevitable adoption of cloud computing and concerns regarding public perceptions of the technology characterized genomics researchers responses. At present, the perception that the field is moving towards a cloud environment is growing:

It is very clear that cloud computing is going to be a component, and probably a significant component, of our future. We're already looking at projects that are going to be using it significantly. I think there's also lots of thought going into, but rather than somebody coming into our data center and us getting them logins to our data center so that they can access data, we potentially get together in the cloud, so we'd upload data into a cloud infrastructure, and then, we would effectively, sort of virtually meet there to carry out our joint analysis or our collaborations there. That, to me, would be something quite desirable that would help. (GR0721)

In anticipation of a cloud environment, some initiatives focus on the establishment of common approaches and practices surrounding the use of cloud computing in genomics research by attempting to streamline how researchers adopt and use cloud technology:

…[The] Global Alliance for Genomics and Health (GA4GH)…[is] an organization that is dedicated to making it easier, more secure, more uniform ways of sharing genome data internationally. So that's the key thing. And of course, the motivation for this, if we don't share the genomes, then we won't understand rare disease. Because if you only see one instance of the genome of a rare disease, you can't generalize what other instances of that rare disease might look like. You have to have access to other genomes and right now, most of the medical institutes around the world have policies to force them to silo their information away. So every, all the genomes that are sequenced in their institute are kept in their own database and no one outside of that institute has access to that. It will never get anywhere with medicine if we continue this way. (GR0731)

Underlying such initiatives is the fear of losing the public trust. In this context, it is feared that news of a breach would be enough to undermine the public’s perception of the technology. Such societal concerns were deemed as contrary, in certain respects, to the benefit of patients. As privacy and cultural concerns drive public opinion and influence policy, that may impede data sharing on a global scale:

Whether or not it's a scandal, because if you can frame it to look bad, it's a problem, even if it's not a problem, or even if it's the right thing to do, if you can frame it as a bad thing to do, then you have a problem because it's just easier to not do something than to do something that might get you in trouble. And so, we have a real challenge around, in the (name of country) particularly, and I think in many other countries, and a lot of it, there's a quite substantial variation in opinion across cultures on these sorts of things, and I suspect in some places, the privacy concerns are a heck of a lot higher and the concerns about dying are a lot lower. So I think trying to put things in a global enterprise, I think, is a real challenge with one ethical, regulatory, legal framework, where it doesn't really fit that way. (GR0715)

However, one researcher shared societal concerns over data privacy and raised them as a basis for not using cloud technology:

People are concerned, if you have medical data, somewhere on the net, an insurance company could steal it, and/or an employer could steal it, and basically make decisions that are unethical because of your use of the cloud. People are little bit hysterical sometimes about data privacy. To date, really, not much has happened, as far as I know. But, people in (name of country) are extremely concerned about that. So for genetic data, there's really no way that people are going to use the cloud anytime soon. (GR0520)

While there existed the sense that the cloud was inevitable, be it for data storage/management issues or on account of pressure from potential cloud providers, there is reluctance over the need to adopt cloud computing. Some even goes as far as to question the relevance of cloud computing in genomics research:

The second aspect of it, which is less important, is that it is not always clear to people why you would bother to do this. They do not see that it is very important. They think that the data are there now. There are big computers there. You can do what you like with them here. Why should you want to put them into a cloud environment. That's unclear to many people. (GR0601)

One researcher viewed cloud computing adoption as a form of hype and questioned its usefulness for his work:

The way I think about things is first, what do I want to do, what goals do I have, and then how do I do it, and there's a lot of hype about cloud computing. That would not be a goal for me. That's a means to an end. And I mean, for the sort of things that we are doing, right now, it's just the case that it's not needed. (GR0520)

#### Cloud service providers

For large cloud service providers, however, genomics research does not so much present a monetary opportunity than an opportunity to play a significant role in an emerging field requiring the organization of sensitive data:

I think some saw an opportunity and I don't think it's necessarily a monetary opportunity. I think the amount of money that we are making from our genomic contracts is growing, it's enough to break even. I don't think there's a profit center for us at all, especially not compared to advertising, which brings us billions. I think there's something of a corporate culture whereby we're very much interested in using data, in organizing data, in using data to solve practical problems. And so we have all these little projects around (name of company) and things you wouldn't expect …The common theme is that we can find ways to use data to solve a practical problem in the world. And I think there’s enough people who are interested. There's a lot of support among executives for starting up a project in helping with the life sciences. (CSP0527)

Their participation in initiatives such as the GA4GH falls in line with their refinement of cloud technology in accordance with the development of common standards deemed necessary for ethical data sharing:

The community is interested in standards for interoperability and there is this international consortium called the Global Alliance for Genomics and Health, which is actively trying to promote standards for policy, ethics, legal, security and also data representation and programmatic access to genomic resources. And so we find that within the community, there's a lot of interest and belief that the future is to have standards for how to access genomic information, how to represent it and not have to use the current model where accessing data means step 1, download it somewhere and then step 2, use command line tools to work with it. The idea is once you have, in the world today, there's already probably 100 petabytes of genomic information. So that order of magnitude, a lot of it is private data, if not publicly shared, but obviously, that would be very big to download anywhere. Even to copy it from one cloud to another cloud, that's a lot of data to move. So the community states that in the future, the future is really to have programmatic standards for how to access the data where it resides. (CSP0612)

#### Patient advocates

Pressure from patients for increased data sharing is a significant reason for which patient advocacy organizations put pressure and undertake initiatives to facilitate data sharing. Patients view data sharing as essential to accelerate the development of treatments and diagnostic tools for their diseases:

To be honest, I think what makes that possible is the urgency that our families have and the different risks/reward calculus that our communities are willing to tolerate. Pushing the ethical/legal envelope around this. I think companies and governments and agencies tend to be a lot more conservative to what they perceive is their exposure in the world of privacy, etc. That's something that we don't have the luxury of when we're working for families who want and need treatment now. And so that's been a very interesting, sort of underlining dynamic for all of our thinking about developing the resource technically, but also thinking about our access policy and what we're pushing for in terms of open sharing of genomic data. (PA0729)

At the same time, patients and families expressed mistrust regarding large data collection agencies and perception that their data will be used for purposes other than research:

Yeah, I think the public understanding is low on this. I think they would be surprised. I think there's a bit of an education piece too. One is to educate people about the importance of commercial cloud structure and the fact that your data is probably being held by (name of cloud service provider 1) or (name of cloud service provider 2) and that's ok, and understanding why those companies are involved. I think there's the big leap there. I think most people are concerned about privacy. Actually, most people who contribute data, and genetic data, are doing so because it's very altruistic for the most part. But they want to have an impact on other people, they want their data to make a difference for future generations or even current patients and I think they'd actually be shocked at how little is actually shared. So I think the public reaction would actually be concern, when people give tissue or give genetic data, they're assuming that people are going to use it and share it and I think if they knew actually how often pathology stuff got tossed out, they'd be horrified. So informed consent is a big thing, portable consent is a big thing (PA0303)

Part of what influences cloud computing adoption stems from efforts to normalize its use in genomics research in an effort to increase public trust in the technology and facilitate data sharing. As public perceptions over cloud technology and data privacy shape policies and regulations, the development of standards provides a means of navigating policies and regulations, which are not suited for the purposes of genomics research. Still, concerns over public trust, permeate the field and shape efforts in anticipation of a cloud environment.

## Discussion and Analysis

**Fig 2:**
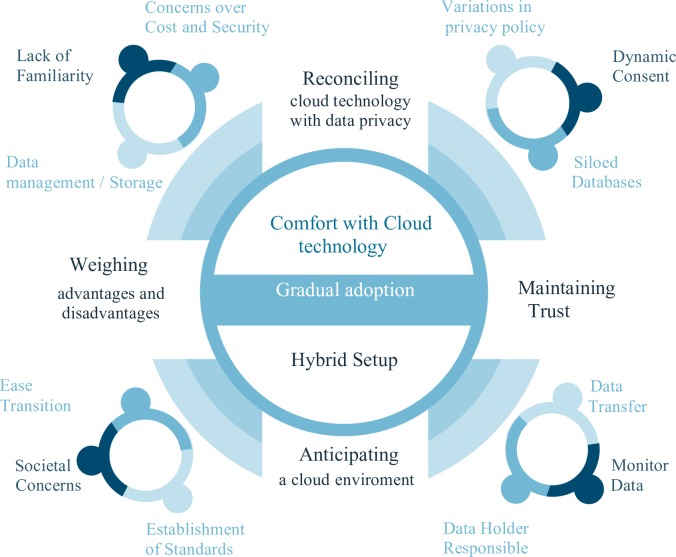
Cloud computing adoption in genomics research.

This study aimed to understand how ethical and legal issues arising in cloud computing shape its use and adoption in the field of genomics research. More specifically, we sought to understand how genomics researchers, patient advocates and cloud service providers manage these various ethical issues and how that shaped the process of cloud computing adoption in the field (Fig 2). The results indicate that, at present, except for large, international genomic projects, there is minimal cloud computing use and adoption in genomics research. In part, researchers tend towards gradual adoption of the technology through hybrid models, keeping their data stored in a local environment and using the cloud for computation. Researchers may also “try-out” cloud technology as a form of gradual adoption. While cloud computing has the potential to mitigate the growing problem of data storage, reluctance to adopt cloud technology persists, and some even question its relevance for genomics research. The advantages associated with cloud computing were measured against ongoing cost and security concerns over the loss of control of data, particularly the need to protect the privacy of patient data. Such concerns translate into increased monitoring costs when installing cloud technology. Lack of familiarity tends to be attributed by some to researchers’ anxiety with cloud technology, prompting them to adopt an overly-cautious attitude.

In an effort to put forth cloud computing as a tool for genomics research, they adapt their services to ease the transition towards cloud technology adoption include the development of pre-configured platform resources facilitating and promoting researchers use of cloud service provider’s infrastructure, characterized as a “partner ecosystem”. The field is thus taking form around the need to increase trust between infrastructure providers and genomics researchers, who remain legally responsible over the data, even as they are limited in their ability to track large cloud service providers’ use of that data.

While an important aspect that was raised in efforts to facilitate cloud adoption relates to the de-siloing of databases, variations in privacy policy regulations among jurisdictions persist alongside public skepticism with cloud technology. The elaboration of common standards aimed at establishing a frame of reference through which to guide researchers through siloed databases is a redefinition of data privacy centered on increased patient control over the use of their data as well as on the notion of multi-stakeholder data ownership. Such a redefinition stems in large part from the patient advocacy community within which alternative consent mechanisms are being promoted to address identifiable genomic data. Thus, the development of alternative consent mechanisms, while empowering patients, also represents an attempt to circumvent institutional and jurisdictional hurdles that tend to restrict data access.

## Conclusion

We drew on five dimensions of DoI theory adapted to address the ethical, legal and social issues in decisions to adopt and use cloud technology. While cloud technology is touted as a necessary tool in advancing genomics research, researchers remain reluctant to immediately adopt it. Concerns over cost and security are ongoing, even though pre-configured platform resources have been developed to facilitate the use of the technology for genomics researchers. The latter remain constrained to trust their cloud service provider to monitor their data, through multiple jurisdictions. Genomics researchers remain legally responsible for their data stored in the cloud. This prompts them to resort to additional security measures, thereby increasing the cost of cloud technology use. While this situation constrains cloud computing adoption, efforts to increase patient control over the use of their data provide an alternative to reconcile data access and data privacy by circumventing institutional and jurisdictional hurdles that form siloed databases.

## Limitations of the Study

Due to the time constraints, certain limitations characterize this study. First, we did not transcribe interviews verbatim, avoiding the pitfalls of transcription, as the latter involves a subjective process in spite of the assumption of transcription as raw data [33, 34]. Second, while interview data was confronted with relevant documents in the course of the data collection process, we were not able to conduct any formal analysis of that documentation. Third, we did not resort to member checking, for fear that it would jeopardize the internal validity of the study given the risk that interviewees might retroactively change their perspective [35, 36].
